# TPC2: From Blond Hair to Melanoma?

**DOI:** 10.3390/cancers16234065

**Published:** 2024-12-04

**Authors:** Carla Abrahamian, Lina Ouologuem, Rachel Tang, Thomas Fröhlich, Karin Bartel, Christian Grimm

**Affiliations:** 1 Walther Straub Institute of Pharmacology and Toxicology, Faculty of Medicine, Ludwig Maximilians University, 80336 Munich, Germany; carla.abrahamian95@gmail.com (C.A.); rachelfaitht@outlook.com (R.T.); 2 Department of Pharmacy, Ludwig Maximilians University, 80539 Munich, Germany; lina.ouologuem@cup.uni-muenchen.de (L.O.); karin.bartel@cup.uni-muenchen.de (K.B.); 3 Laboratory for Functional Genome Analysis LAFUGA, Gene Center, Ludwig Maximilians University, 80539 Munich, Germany; frohlich@genzentrum.lmu.de; 4 Immunology, Infection and Pandemic Research IIP, Fraunhofer Institute for Translational Medicine and Pharmacology ITMP, 80333 Munich, Germany

**Keywords:** TPC, TPC2, TPCN2, two-pore channel 2, lysosome, melanosome, melanoma, melanocyte

## Abstract

Two-pore channel 2 (TPC2) is expressed in endolysosomes throughout the human body, as well as in melanosomes of melanocytes. Melanocytes produce pigment, i.e., melanin, which determines hair and skin color but also protects from UV light. Extensive exposure to UV light is one of the major risk factors for the development of melanoma, which develops from pigment-producing cells, i.e., melanocytes. In recent years, several human TPC2 single nucleotide polymorphisms have been identified to increase the likelihood of carriers presenting with blond hair and hypopigmentation. These variants were all characterized as gain-of-function versions of TPC2. Vice versa, the loss of function of TPC2 increases melanin production and reduces cancer hallmarks such as proliferation, migration, invasion, tumor growth, and metastasis formation. The activity of TPC2 is controlled in a complex manner, with several endogenous ligands as well as a number of interacting proteins being involved. We will discuss here the role of TPC2 in pigmentation and its potential to impact melanoma development and progression and highlight recent findings on Rab7a as an enhancer of TPC2 activity.

## Simple Summary

Two-pore channel 2 (TPC2) is a protein mediating Ca^2+^ and Na^+^ flux across the membranes of intracellular vesicles, including melanosomes. Melanosomes produce pigment (melanin), giving hair and skin their color. Blond or red hair and fair skin, in particular in combination with extensive UV light exposure, are *bona fide* risk factors for melanoma development. While TPC2 gain-of-function variants decrease melanin production, resulting in an increased likelihood for carriers to have light/blond hair, loss of TPC2 function increases melanin production. Here, we will discuss recent evidence for TPC2 and its effector Rab7a as proteins involved in melanin production and their implications for melanoma development.

## 1. Introduction

In Greek, Roman, and Norse mythology, the goddesses of love, beauty, and fertility, Aphrodite, Venus, and Freyja, as they were called, respectively, were described as having blond or golden hair. Greek heroes in Homer’s Iliad, like Menelaus, the king of the Spartans, or Achilles, the central character in the Iliad, were portrayed as having blond hair. Whether Aphrodite, Boticelli’s ‘Birth of Venus,’ or Marylin Monroe, blond hair seems to have exerted a certain fascination for centuries, particularly in Western societies.

The Romans associated blond hair mostly with Germanic tribes, and indeed, blond hair is well known to be more common in Northern European countries than anywhere else in the world. Of note, blond hair seems to have evolved more than once [1,2], not only in Northern Europe but also, seemingly independently and far away from Europe, on the Solomon Islands (Melanesia), where mutations resulting in blond hair might have arisen 5000–30,000 years ago [1,3]. Recent genetic analyses also suggest that the Kalasha, or Kalash, people of Northern Pakistan (Chitral District), who often have pale skin and light-colored eyes, and sometimes also blond hair, might not be descendants of Europeans, i.e., of Greek soldiers who came to that region with Alexander III of Macedon, commonly known as Alexander the Great, as originally thought. Nevertheless, the European descent or admixture theory is still a matter of debate, and the Kalash themselves claim to be descendants of Alexander the Great and the ancient Greeks [4,5,6,7]. Like the Kalash, the Nuristanis, an ethnic group native to the Nuristan Province of northeastern Afghanistan, and other Eurasian populations can be encountered having pale skin, light-colored eyes, and blond hair.

In the last two decades, scientists have discovered a plethora of genes affecting pigmentation and melanin production with effects on skin and hair color in mice and humans, such as TYRP1, TYR, KITLG, OCA2, SLC45A2, SLC24A4, TYR, ASIP, EDNRB, MC1R, IRF4, and also TPCN2, the gene encoding the endolysosomal cation channel TPC2 [2,3,8,9]. Recent GWAS studies even claim up to 200 genetic variants are associated with multiple hair colors on the spectrum of blond to black [8]. TPC2 gain-of-function (GOF) polymorphisms are associated with blond hair color, hypopigmentation, and albinism [9,10,11,12]. Vice versa, knockout, knockdown, or pharmacological inhibition of TPC2 results in increased melanin production and decreased cancer/melanoma proliferation, migration, invasion, tumor growth, and metastasis formation [13,14].

Importantly, blond and red hair are also associated with an increased risk for skin cancer, in particular melanoma. In the USA and the EU, every year, between 80,000 and 100,000 individuals are diagnosed with melanoma, and between 8000 and 16,000 individuals die each year from the disease in the USA and in the EU, with numbers on the rise. Improvements in diagnosis, in particular, early diagnosis and better therapeutic options, are urgently needed.

In the following, we will discuss the latest on TPC2 and its role in melanoma, as well as its functional interaction with the oncogene Rab7a. Moreover, we will provide novel proteomics data comparing melanoma cell knockouts of TPC2 and TRPML1, the latter one being another endolysosomal cation channel highly expressed in melanocytes, proposed to play a role in metastatic melanoma [15,16].

## 2. Human TPC2 Polymorphisms and Melanin Production

TPC2 plays a crucial role in melanin production and pigmentation in melanocytes and melanoma cells [13,14,17]. Several human GOF single nucleotide polymorphisms (SNPs), TPC2^M484L^, TPC2^G734E^, and recently TPC2^R210C^, are associated with hypopigmentation, blond hair color, and dominant albinism [9,10,11,12]. Studies have revealed distinct functional alterations in these SNPs, with TPC2^M484L^ [10] and TPC2^R210C^ [12] demonstrating shifts in the dose–response curve of the endogenous TPC2 agonist PI(3,5)P_2_ (phosphatidylinositol 3,5-bisphosphate), resulting in lower potency and efficacy of the WT compared to the respective SNPs, while TPC2^G734E^ exhibits a reduced response to inhibition by ATP. All three SNPs are, therefore, classified as GOF variants, albeit based on different mechanisms. In a cohort of >100 individuals, Chao et al. (2017) [10] observed that homozygous M484L or G734E polymorphism carriers had an increased likelihood to present with light hair or blond hair versus dark hair or brown/black hair color, corroborating data from Iceland and the Netherlands published earlier by Sulem et al. (2008) [9]. Furthermore, Wang et al. (2023) [12] reported a case of a Chinese proband being homozygous for TPC2^R210C^ with hypopigmented skin and hair. The parents of the proband, by contrast, did not show variation at that position, and both presented with black/dark hair, suggesting TPC2^R210C^ as a de novo variant in the affected child. To further demonstrate a direct link to TPC2, the authors generated knock-in mice for the equivalent position in the murine gene, which displayed similar hypopigmentation. The expression of TPC2 in both lysosomes and melanosomes suggests different functions relevant to melanoma development, one being decreased melanin production, likely due to luminal pH reduction resulting in the reduction of tyrosinase activity, the key enzyme in melanin synthesis with a pH optimum at around 6.8. In sum, evidence for TPCN2 being an important gene determining hair color is accumulating and backed up by recent functional data [9,10,12].

## 3. Fair Skin, Light Hair Color, and Increased Melanoma Risk

Fair skin, light hair color, or blond/red hair are *bona fide* risk factors for melanoma development. Other risk factors include extensive UV light exposure, a history of sunburn, and many moles (nevi), especially asymmetric ones with irregular/uneven borders, color variation, or evolving changes in diameter, color, or shape. Additionally, age and genetics are also risk factors, e.g., mutations in CDK4, CDKN2A (involved in cell cycle arrest and melanocyte senescence), and genes, which are major determinants of hair/skin pigmentation, such as MC1R, TYR, or ASIP. Thus, variations in the melanocortin 1 receptor (MC1R), which can produce a fair-skinned and red-haired phenotype, are well-established drivers of melanoma risk in red-haired individuals but may also increase the risk in darker-haired or darker-skinned individuals [18,19,20]. ASIP and TYR pigmentation variants were shown to associate with cutaneous melanoma (CM) and basal cell carcinoma (BCC) by Gudbjartsson et al. (2008). In that study, OCA2, KITLG, SLC24A4, and TPCN2, which are all associated with blond hair or blue eyes, did not show a nominally significant association with the risk of CM or BCC. By contrast, a later study found an association of OCA2 variations with BCC risk and also confirmed increased melanoma risk for variations in TYRP1, SLC45A2, and ASIP and increased SCC risk (squamous cell carcinoma) for TYR and ASIP [21]. In addition, interactive effects of MC1R and OCA2 on melanoma risk were reported [22]. In sum, although not all genetic variants underlying pigmentation traits may confer a detectable risk for skin cancer [18], fair skin and blond/red hair seem to be risk factors for melanoma development, in particular when combined with other risk factors such as extensive UV light exposure. In line with this, skin cancer and melanoma are known to be more common in white compared to colored individuals [19,23]. Albeit more rare, skin cancer has been found to be more aggressive in African American communities in the United States [24].

## 4. TPC2, Its Regulator Rab7a and Its Functional Relative TRPML1 in Melanoma

Knockout (KO), knockdown (KD), or pharmacological inhibition of TPC2 in different cancer cell lines, including melanoma lines, results in reduced migration, invasion, and proliferation of cancer cells in vitro and tumor growth and metastasis formation in vivo [14,25,26]. The endolysosomal cation channel TPC2 is one out of two mammalian TPCs with predominant subcellular expression in LE/LY in addition to melanosomes, while TPC1 resides more in early endosomes (EE) and has no clear association with hair color or pigmentation. TPC2 activity is controlled by PI(3,5)P_2_, a major phosphoinositide constituent of LE/LY membranes, by NAADP (nicotinamide adenine dinucleotide phosphate), which reportedly binds to TPCs indirectly via auxiliary proteins such as JPT2 or Lsm12 [27,28,29], and by ATP, which likewise acts on TPCs indirectly via mTOR (mammalian target of rapamycin), blocking the channel. Recently, Abrahamian et al. (2024) found that the small GTPase Rab7a is a strong enhancer of TPC2 channel activity [30]. Rab7a itself is known as an oncogene with reported roles in cancer and melanoma [31]. Abrahamian et al. (2024) demonstrated that the effect of Rab7a on TPC2 activity was found to be independent of the respective agonist used for activation. Rab7a also shows conspicuous overlap with TPC2 functions and features [30]. Both TPC2 and Rab7a localize to LE/LY and melanosomes [32] and control trafficking and vesicular transport in the late endolysosomal pathway, e.g., trafficking and degradation of EGF/EGFR and adhesion molecules [25,33,34,35,36]. Moreover, both play key roles in cell proliferation, growth, migration, and autophagy. In analogy to TPC2, Rab7a knockdown in NPC1 cells exacerbates cholesterol accumulation [37,38,39], and in melanoma cells, Rab7a levels are significantly higher than in healthy skin melanocytes, impacting melanoma proliferation and invasion [31,38,40]. Abrahamian et al. (2024) showed that loss of Rab7a can be rescued by TPC2 overexpression or overexpression of the GOF variant TPC2^M484L^ with or without the lipophilic small molecule agonist of TPC2, TPC2-A1-P, or with the agonist alone. Vice versa, loss of TPC2 is barely compensated by overexpression of Rab7a^WT^ or the GOF mutant Rab7a^Q67L^. These findings suggested that Rab7a is an “effector” of TPC2 but not vice versa (with “effector” being defined here as an upstream molecule (Rab7a) interacting with a downstream target (TPC2) to increase its activity) and that effects of Rab7a on proliferation, migration, and invasion of tumor cells are mediated predominantly by TPC2. This suggests that effects seen in other disease pathologies due to Rab7a knockout or modulation may likewise be mediated by TPC2 [30].

Another endolysosomal cation channel shown recently to exert effects on proliferation, migration, and invasion of tumor cells, including melanoma, is TRPML1 [15,16,41,42,43]. Data about TRPML1 in melanoma are somewhat controversial. Kasitinon et al. (2019) demonstrated that the loss of TRPML1 in melanoma impairs proliferation in vitro and reduces melanoma tumor growth in vivo without affecting normal melanocytes via upregulation of MAPK and mTORC1 signaling, overcoming proteotoxic stress [15,16,41,42,43]. However, Du et al. (2021) showed that Zn^2+^ release mediated by lysosomal TRPML1 triggers rapid, mitochondria-mediated, non-apoptotic cell death in metastatic melanoma. Treatment of melanoma cell lines, specifically M12 and MeWo lines, with TRPML1 agonists MLSA5 or ML-SA8 triggered cell death. In contrast, suppression of TRPML1 using ML-SI4 or ML-SI3 did not have any effect [15,16,41,42,43]. These findings challenge not only the earlier report by Kasitinon et al. (2019), claiming that TRPML1-deficient melanoma cells exhibit decreased survival, proliferation, and tumor growth [15,16,41,42,43], but also several other reports on the role of TRPML1 in cancer, either claiming that cancer cells are vulnerable to TRPML1 inhibition or downregulation [41,44,45,46]. Thus, Frey et al. (2023) proposed that loss of TRPML1 function is associated with reduced cancer migration, adhesion, and spheroid formation in vitro and reduced cancer cell dissemination in vivo [42]. Silencing or pharmacological inhibition of TRPML1 in aggressive triple-negative breast cancer reduced invasion and tumor growth in vitro and in vivo [44] and diminished the proliferation of cancer cells expressing oncogenic HRAS [45]. By contrast, Qi et al. (2021) showed that activating TRPML1 with the agonists ML-SA5 or MK6-83 triggers cell death of a number of cancer cells by evoking autophagic arrest and subsequent apoptotic response and cell cycle arrest [47]. In HCC cell lines, Siow et al. (2022) reiterated this duality of TRPML1 in cancer. Thus, activation of TRPML1 was found to lead to cancer cell death by impairing mitochondrial function and might, therefore, be beneficial in cancer therapy, while in the same cells, TRPML1 loss-of-function leads to mitophagy defects, which, despite impairing mitochondrial function, do not induce cell death but instead result in a reduced rate of proliferation [15,41,43,44,45,46].

These discrepancies in results may, at least in part, have to do with the fact that TRPML1, in contrast to TPCs, can not only release Ca^2+^ and Na^+^ but also Zn^2+^, Fe^2+^, and other metal ions, adding an additional layer of complexity regarding the physiological outcome [48,49].

**Figure 1:**
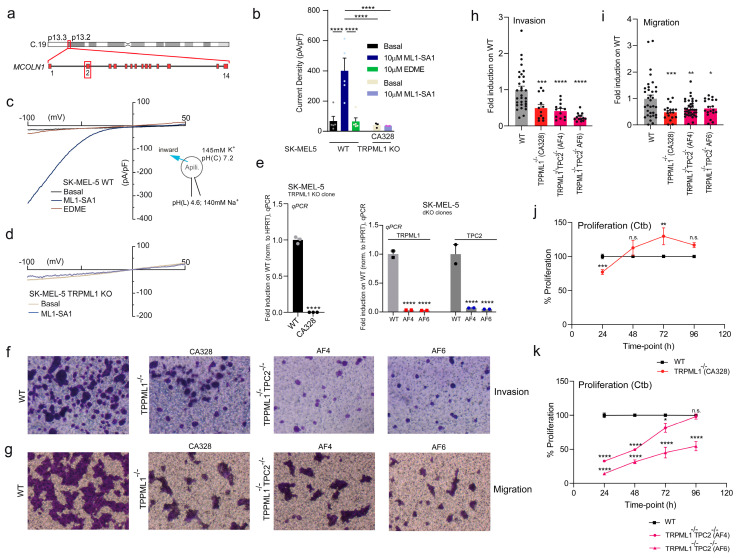
(**a**) CRISPR/Cas9 gene editing strategy for the human *MCOLN1* gene in the SK-MEL−5 melanoma cell line (described in detail in Rühl et al. (2021) [41]). The generation of TPC2 KO and double KO cells was described in Abrahamian et al. (2024) [30]. (**b**) Statistical analysis of the average current densities measured in endolysosomal patch-clamp experiments for the SK-MEL−5 WT and TRPML1 cells. Each dot on the bar diagram represents a single value current density measured from one endolysosome. The dataset was tested for statistical significance using a one-way ANOVA test followed by Tukey’s post-test. (**c**,**d**) Representative current densities obtained from apilimod-enlarged endolysosomes showing basal, ML1-SA1 (TRPML1-specific agonist [50]) in the SK-MEL−5 WT (**c**) and TRPML1-KO (**d**) and EDME (TRPML1-selective antagonist, [41]) in WT cells. (**e**) qPCR validation of the SK-MEL−5 TRPML1 KO clone (CA328) and statistical analysis determined by a Student’s t-test and TRPML1/TPC2 dKO clones (AF4 and AF6), showing reduced transcript levels for TRPML1 (single KO) and both channels (dKO) and statistical analysis assessed by using two-way ANOVA followed by Bonferroni multiple comparisons test. (**f**) Transwell chambers migration experiment performed, indicating a reduction in migration for the TRPML1-KO line and significantly fewer migratory cells for the dKO as compared to WT. (**g**) Transwell chamber invasion assay (coated with matrigel) shows a significant reduction in the SK-MEL−5 TRPML1 and dKO line compared to WT cells. (**h**,**i**) Statistical analysis for the invasion assay (**h**) and migration (**i**) determined by one-way ANOVA. (**j**) Ctb proliferation assay monitored over 96 h shows a reduced proliferative phenotype for 24 h only for the TRPML1-KO cells. (**k**) Ctb proliferation assay in the two dKO clones showed a significant reduction in proliferation over different time points compared to the WT SK-MEL−5 line. Statistical significance for the proliferation experiments was carried out using two-way ANOVA, followed by the Bonferroni multiple comparisons test. * *p* < 0.05, ** *p* < 0.01, *** *p* < 0.001, **** *p* < 0.0001.

We have generated TRPML1 KO SK-MEL-5 melanoma cells in addition to previously generated KOs for Rab7a and TPC2 in SK-MEL-5 cells [30] using CRISPR/Cas9. Additionally, we generated TPC2/TRPML1 dKO lines. We validated these new KO cell lines by using qRT-PCR and endolysosomal patch-clamp electrophysiology as described previously for TPC2 [14,41] (Figure 1a–e). Similar to TPC2 KO, TRPML1 KO showed a strong reduction in the migration and invasion of SK-MEL-5 cells. Yet, the effect on proliferation was less prominent in TRPML1 KO cells in contrast to TPC2 KO, a phenomenon that was already observed in other melanoma and also hepatocellular carcinoma cell lines before [14,16,26,42]. By contrast, the dKO of TRPML1 and TPC2 showed strong effects in all three parameters: migration, invasion, and proliferation (Figure 1f–k), but no obvious synergism.

**Figure 2:**
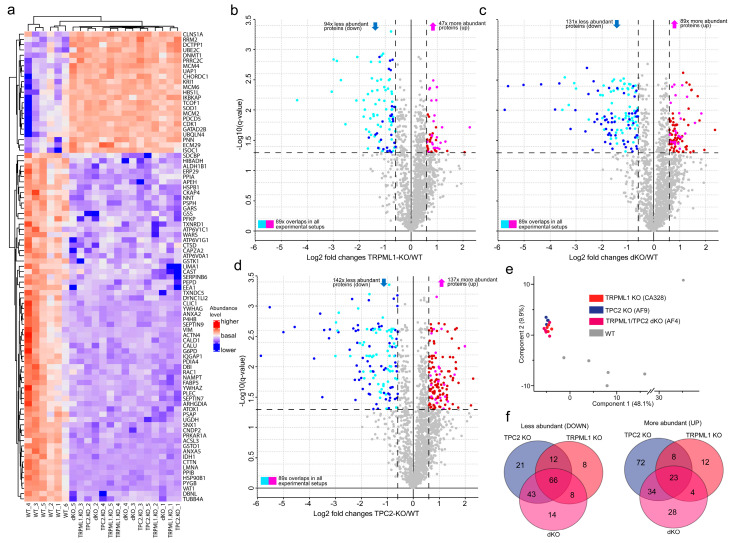
(**a**) Heat map displaying label-free quantification intensities for the 89 overlaps (proteins) in all three experimental setups with dendrogram-based grouping according to experimental conditions. (**b**–**d**) Volcano Plot analysis was performed for three experimental subsets: (**b**) TRPML1 KO vs. WT, (**c**) dKO vs. WT, and (**d**) TPC2 KO vs. WT, displaying all differentially abundant proteins in each, resulting upon filtering based on p.adj. < 0.05 and |Log2 fold change| > 0.6. Blue shades = less abundant proteins in KO, and red shades = more abundant proteins in KO. Lighter shades display the 89 overlapping proteins in all three subsets (as mentioned in the heat map above). (**e**) Principal component analysis demonstrating sample-wise grouping according to experimental conditions. (**f**) Venn diagrams of less and more abundant proteins in all three experimental setups, as well as the total of 89 overlaps i.e., 66 less abundant and 23 more abundant entries. The entire analysis was performed using MaxQuant Perseus v.1.6.5.0.

Remarkably, when performing proteomics analysis of the three genotypes i.e., of the TPC2 KO, TRPML1 KO, and dKO SK-MEL-5 lines, a strong overlap in proteins that were down- or upregulated in the single and dKO genotypes as compared to WT control was found (Figure 2a–f). Thus, the KOs were all tightly clustered (Figure 2e) compared to WT. Many proteins that were upregulated/downregulated in either single KO were also upregulated/downregulated in dKO samples (Figure 2a–d,f). In total, 66 identical proteins were downregulated in TPC2KO, TRPML1KO, and dKO alike, and 23 proteins were upregulated in all three groups (Figure 2f). Thus, these data not only suggest a difference in the proteomes of WT versus KOs and dKOs but also several common regulations between the KO proteomes. Proteins found to be downregulated in all three genotypes include proteins essential for lysosomal function, such as the V-type proton ATPase subunits ATP6V1C1, ATP6V1G1, and ATP6V0A1, or cathepsin D (CTSD); proteins involved in intracellular trafficking or vesicle transport, such as sorting-nexin 1 (SNX1) and EEA1 or VAT1; proteins with reported roles in cancer, including melanoma, such as Rac1 (Ras-related C3 botulinum toxin substrate 1), the Ras GTPase-activating-like protein IQGAP1, CKAP4, APEH, HSP90B1, or PYGB; proteins involved in degradation in lysosomes, such as PSAP (Prosaposin); or proteins involved in cell migration (Cortactin, CTTN). Others, e.g., proteins involved in cell cycle arrest and proliferation such as MCM proteins (MCM2, 4, 6), proteins involved in DNA methylation (DNMT1), proteasomal protein degradation (Ubiquilin4, UBQLN4), or apoptosis regulation such as programmed cell death protein 5 (PDCD5) and SOD1 were upregulated in all KO samples compared to WT. Interestingly, these data also show that the overlap of proteins being up- or downregulated is higher between TPC2 KO and dKO as compared to TPC2 KO and TRPML1 KO or TRPML1 KO and dKO. This higher similarity of TPC2 KO with dKO cells is further corroborated by the functional data shown in Figure 1 (proliferation, migration, and invasion data) and the data published previously for TPC2 KO [30].

A key cellular process in which TPC2 and TRPML1 have been proposed to play important roles is autophagy. Autophagy is the cellular process involved in the recycling and degradation of damaged cellular components, which is essential for the maintenance of cellular homeostasis [37,51,52]. However, the role of autophagy in cancer is controversially discussed. Thus, it is reported that during the early stages of cancer initiation, autophagy impacts tumor suppression through the elimination of damaged organelles and by reducing the production of reactive oxygen species (ROS). In later stages of cancer progression and metabolic stress, autophagy reportedly acts as a pro-metastatic mechanism, promoting the survival of cancer cells [53,54]. TFEB (transcription factor EB) plays a central role in the regulation of autophagy and lysosomal function [55]. It belongs to the microphthalmia family of evolutionarily conserved members in the vertebrates, also including TFEC, TFE3, and MITF (microphthalmia-associated transcription factor) [56], whereby the latter one has recently been shown to play a key role in TPC2/Rab7a-driven effects in melanoma cells [30].

TRPML1, TPC2, and Rab7a are all key players in autophagy and are directly or indirectly regulating mTORC1 and autophagy pathways [37,52,57,58,59]. mTORC1, a serine/threonine kinase, phosphorylates TFEB during physiological conditions, inhibiting its activity and translocation to the nucleus. By contrast, when mTORC1 is blocked during stress or nutrient starvation periods, nuclear translocation of TFEB is facilitated and, subsequently, the transcription of lysosomal genes. Rab7a promotes the fusion of lysosomes and autophagosomes, forming autophagolysosomes and contributing to lysosomal biogenesis [59]. During starvation, Ca^2+^ release via TRPML1 leads to mTORC1 suppression, promoting TFEB translocation into the nucleus and autophagy by activation of calcineurin [52,57], and for TPC2, a direct interaction was shown with mTOR. Thus, under cellular stress and high ATP, mTOR phosphorylates and inhibits TPC2 [60]. Chao et al. (2017) showed a reduction in ATP sensitivity for the TPC2^G734E^ GOF variant [10], resulting in the need for higher concentrations of ATP to block TPC2, possibly due to reduced mTOR/TPC2 interaction.

Importantly, while Rab7a acts as an effector of TPC2, the activity of TRPML1 seems not to be affected by Rab7a [30]. Recently, Vestre et al. (2021) proposed instead an interaction of Rab7b with TRPML1. However, functional data for this interaction are missing, i.e., endolysosomal patch-clamp electrophysiology or GCaMP-based Ca^2+^ imaging data to corroborate an effect of Rab7b on TRPML1 channel activity [61]. While Rab7a localizes to late endosomes and lysosomes and controls trafficking between early/sorting endosomes and late endosomes/lysosomes, Rab7b controls vesicular trafficking from endosomes to the TGN. However, neither of the latter two compartments has been shown to express TRPML1 [35,62].

Despite significant differences in their interactomes and functions, TRPML1 and TPC2 exhibit similarities in ion permeabilities (i.e., Ca^2+^ and Na^+^ permeability), and their involvement in cancer, including melanoma, likewise displays several similarities. Thus, there appear to be overlapping effects upon activation or inhibition/knockout/knockdown of either TPC2 or TRPML1. Future research will have to look deeper into the unique and overlapping effects of TPC2 versus TRPML1 modulation in order to fully understand their roles in cancer/melanoma and their potential as drug targets.

## 5. Conclusions

Like in all types of cancer, early diagnosis is key in melanoma as well. Regular skin assessments using artificial intelligence-based systems are increasingly standard and will help to diagnose patients early. In recent years, there has also been progress in pharmacological therapy. For example, major advances in treating stage II, stage III, and stage IV melanoma have been made with immunotherapy e.g., with programmed death-1 (PD-1) inhibitors or the cytotoxic T-lymphocyte-associated molecule-4 (CTLA-4) inhibitor Ipilimumab, which has been shown to shrink melanoma in 10% to 15% of patients. Both PD-1 blockers, Nivolumab and Pembrolizumab, have been shown to shrink melanoma in 25% to 45% of patients with unresectable melanoma. Other approaches include BRAF inhibitors such as Dabrafenib, Encorafenib, or Vemurafenib in melanoma with mutated or activated BRAF genes or MEK inhibitors or combinations thereof [19,63,64,65,66]. As of now, it is unclear whether TRPML1 or TPC2 inhibitors could provide any clinical benefit for melanoma patients. Much more research is needed to mechanistically better understand how TRPML1 or TPC2 inhibitors may impact melanoma development and progression in vivo and, most importantly, what consequences TRPML1/TPC2 inhibition, in particular long-term inhibition, would have. While the temporary inhibition of TPC2 using, e.g., the tetrandrine-derived TPC2 inhibitor, SG094, has demonstrated anti-tumor effects in vivo, with reduced toxicity and improved potency compared to the parent molecule tetrandrine, the long-term consequences of TPC2 inhibition remain speculative [26]. Given the known effects of long-term or permanent loss/inhibition of TRPML1 in humans, resulting in the lysosomal storage disorder (LSD) mucolipidosis type IV with a severe neurodegenerative phenotype and severely reduced life span, TRPML1 inhibition seems not to be a particularly safe option, especially in younger patients. Albeit TPC2 inhibition per se does not result in an obvious neurodegenerative phenotype in knockout mice, long-term inhibition interferes with lysosomal function and intracellular trafficking [33,37,49]. While TPC2 knockout mice show a normal life span, in vitro block or loss of TPC2 seems to enhance lysosomal storage phenotypes caused by other gene defects, e.g., mutations in TRPML1 or NPC1 [39]. By contrast, activation of TPC2 enhances intracellular trafficking, lysosomal exocytosis, and autophagy, thus ameliorating lysosomal storage in different LSDs [37]. Therefore, when evaluating the potential of TRPML1 or TPC2 as novel pharmacological targets for disease therapy, an activation-based disease interference rather than inhibition appears to be the safer option and preferred path forward for the implementation of these ion channels as drug targets.
